# A systematic review of evidence regarding the association between time to mobilization following hip fracture surgery and patient outcomes

**DOI:** 10.1302/2633-1462.67.BJO-2024-0243.R1

**Published:** 2025-07-03

**Authors:** Veena Mazarello Paes, Andrew Ting, James Masters, Mahalia V. I. Paes, Simon Mathew Graham, Matthew L. Costa, On behalf of the HIPCARE investigators*

**Affiliations:** 1 Oxford Trauma and Emergency Care, Nuffield Department of Orthopaedics, Rheumatology and Musculoskeletal Sciences, University of Oxford, Oxford, UK; 2 Kingston Hospital NHS Foundation Trust, London, UK; 3 Queen Mary University of London, London, UK

**Keywords:** Performance indicators, Quality standards, Hip fracture, Fragility fracture, Early mobilization, Weightbearing status, Systematic review, Trauma, hip fracture surgery, hip fracture, patient-reported outcomes, randomized clinical trials, clinical outcomes, retrospective cohort studies, postoperative complications, prospective studies, deep vein thrombosis, 12-item short form survey (SF-12)

## Abstract

**Aims:**

Performance indicators are increasingly used to evaluate the quality of healthcare provided to patients following a hip fracture. In this systematic review, we investigated the association between ‘early mobilization’ after surgery and patient outcomes.

**Methods:**

Evidence was searched through 12 electronic databases and other sources. The methodological quality of studies meeting the inclusion criteria was assessed. The protocol for this suite of related systematic reviews was registered at PROSPERO: ID = CRD42023417515.

**Results:**

A total of 24,507 articles were reviewed, and 20 studies met the inclusion criteria for the review, involving a total of 317,173 patients aged over 60 years with a hip fracture. There were two randomized clinical trials, five prospective studies, and 13 retrospective cohort studies, conducted between January 1981 and June 2022. All but two studies came from high-income healthcare systems. The definition of early mobilization varied across studies and health systems; and weightbearing status was often not reported or ambiguously defined, making formal meta-analysis of the data impossible. Early mobilization (within 48 hours of surgery) was associated with improved outcomes in 29 of the 33 patient-reported outcomes, including improved mobility scores and improved assessments of daily activities of living. A total of 45 out of 51 clinical outcomes derived from hospital records showed a positive association with early mobilization, including reduced rates of postoperative complications, reduced length of acute hospital stay, and lower mortality.

**Conclusion:**

Early mobilization after surgery for hip fracture in older people is associated with improved patient-reported outcomes and reduced length of hospital stay. Standardization of the definition of early mobilization and consistent reporting of weightbearing status would improve future evidence synthesis.

Cite this article: *Bone Jt Open* 2025;6(7):741–747.

## Introduction

Worldwide, the annual incidence of hip fracture is predicted to rise to 6.26 million by the year 2050.^1^ The incidence of hip fracture is increasing, as are the associated years lost to disability and the costs to health and social care systems.^2,3^ After a hip fracture, patients have a one-year mortality rate of 25%, and experience a permanent reduction in their health-related quality of life similar to having a stroke.^4^

In the year 2018, the global Fragility Fracture Network led a call to action based upon the premise that patients with fragility fractures, and hip fracture patients specifically, require input from different types of healthcare professionals in a time-sensitive manner.^5^ For example, the early mobilization of patients after hip fracture surgery requires surgeons, therapists, and nurses to work together to facilitate unrestricted weightbearing mobilization on the day of or the day after surgery, in order to avoid complications and accelerate rehabilitation. In response, many countries have adopted targets, or performance indicators, which include early mobilization.^6^

We performed a systematic review to investigate the evidence that early mobilization after surgery for hip fracture improves patient outcomes.

## Methods

This study is part of a suite of systematic reviews evaluating the use of performance indicators in patients with fractures (registered at the International Prospective Register for Systematic Reviews (PROSPERO).^7^ The review is reported according to the PRISMA guidelines.^8^

### Search strategy

A comprehensive search strategy without age, time period, or language restrictions was iteratively derived with input from information specialist and hip fracture experts (Supplementary Material). In total, the 12 bibliographic databases searched for studies published from database inception to 25 April 2023 were: MEDLINE; Embase; EMCARE; Ovid Global Health; CINAHL; the Cochrane Database of Systematic Reviews; Cochrane Central Register of Controlled Trials; Scopus; Web of Science; WHO Global Index Medicus; CRD NHS Economic Evaluations Database (to 31 March 2015); and the INAHTA Health Technology Database. The search involved using relevant index terms and free-text terms, synonyms, and phrases in the title and abstract fields for relevant papers on performance indicators, quality indicators, health care/quality improvement, and fractures, trauma, injuries, or injury, in order to meet the aims of the protocol. All references were exported into EndNote 20 reference manager (Clarivate, USA), and duplicates were removed using the Bramer method.^9^ Through a process of snowballing, websites, personal databases, and citations were then searched up to 5 January 2025 for additional records.

### Study selection

To ensure a high level of agreement between reviewers, and to minimize any reviewer-related biases, a subset of articles was piloted for independent double review among all authors at each stage of the review process (i.e. title and abstract screening, data extraction, and quality assessment) and results were compared. Titles and abstracts were then independently screened by AT, MVIP, and VMP. Articles appearing to meet this review’s inclusion criteria were retrieved for full-text review, and details of these studies were recorded in pre-piloted spreadsheets with reasons for excluding studies. Foreign-language papers were translated using Microsoft/Google translator, and native speakers were contacted if anything was unclear. Uncertainty about inclusion criteria and disagreements were resolved by discussion among all authors.

### Quality assessment

The Mixed Methods Appraisal Tool (MMAT) v. 2018 was used to assess the methodological quality of included studies.^10^ All authors were trained to use the tool, and a sample of studies was piloted for quality assessment. Two reviewers (AT, VMP) independently assessed the quality of all included studies. Any disagreement between the reviewers over the risk of bias of an included study was resolved by discussion at team meetings. The quality assessment stage underpinned the context of the synthesized findings, but was not used to exclude studies.

### Data extraction and analyses

Two reviewers (AT, VMP) independently extracted data from studies meeting the inclusion criteria to ensure agreement and consistency in data extraction and reporting. Any disagreement was resolved by further discussion among AT, MLC, and VMP.

### Data synthesis

The definition of early mobilization varied across studies and health systems, and weightbearing status was often not reported or ambiguously defined, making formal meta-analysis of the data impossible. Therefore, a narrative synthesis of the data was undertaken and the association between early mobilization and patient outcomes presented descriptively.

## Results

**Fig. 1:**
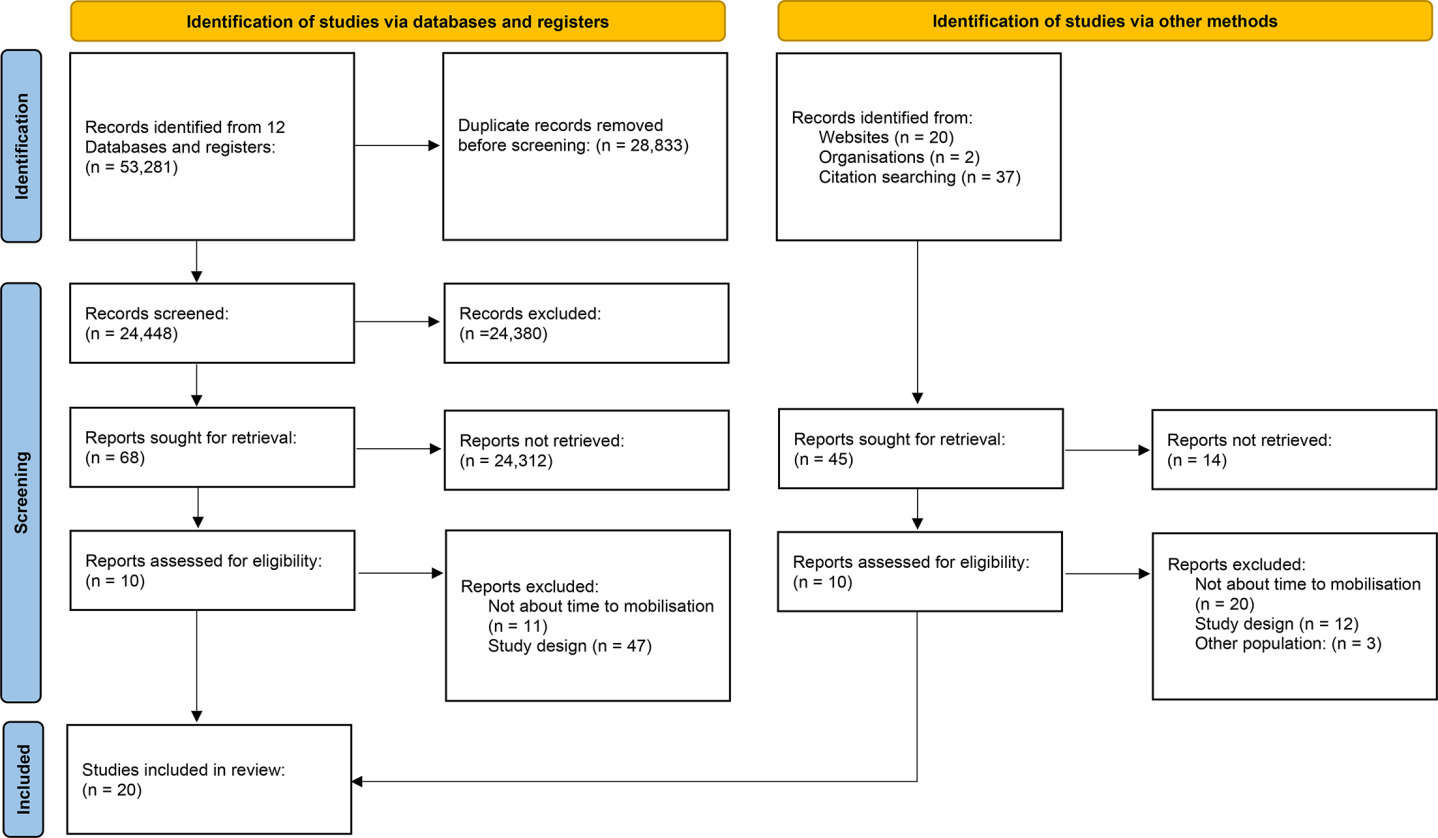
PRISMA systematic search for evidence on the relationship between early mobilization and clinical/patient outcomes in hip fracture. This is a PRISMA 2020 flow diagram for new systematic reviews, which included searches of databases, registers, and other sources.

A total of 24,448 articles were identified from searching 12 electronic databases in April 2023. After screening, 68 full-text articles were reviewed (including two non-English-language papers). Full texts of 59 additional studies identified from reference lists and other sources were also reviewed. A total of 20 studies met the inclusion criteria (Supplementary Material). A PRISMA flow diagram summarizes the literature search strategy (Figure 1).

The articles were heterogenous in terms of health systems, study designs, and study periods. Outcomes were reported in a total of 317,173 older patients (average age 80+ years; approx. 72.5% female) from two randomized clinical trials (RCTs), and five prospective and 13 retrospective cohort studies, conducted between January 1981 and June 2022. All but two studies came from high-income healthcare systems, with one from China and the other from Turkey. The methodological quality of the included studies was high (n = 2), intermediate (n = 4), and low (n = 14).

The definition of ‘early’ mobilization was variable. Three articles defined early mobilization as “within 24 hours of surgery”,^11-13^ seven articles defined early mobilization as the “day of or the day after the surgery”,^14-20^ and a further six articles “within 48 hours of surgery”.^21-26^ However, the other four studies defined early mobilization as between two and seven days after surgery.^27-30^ What constituted ‘mobilization’ also varied from “bed to chair” through “time to walk 3 m”. The description of walking aids was incomplete, but most studies indicated that walking aids were available as required. Weightbearing status was not defined in 13 of 20 studies, and defined ambiguously in others with the use of terms such as “partial weightbearing” and “weightbearing as tolerated”. Similarly, a wide variety of both patient-reported and clinical (based on medical records or routine data) outcomes were reported (summary of extracted data and quality assessment in the Supplementary Material).

### Objective outcome measurement

The effect of ‘early mobilization’ on subsequent mobility scores in acute care was assessed by 12 studies using a variety of measures including the Timed Up and Go; and three and five metre walking tests (Table I). Ten of the studies showed that early mobilization (within 48 hours of surgery) had a positive association with improved early postoperative mobility in hip fracture patients.^11,13,17,18,20,22,24,26,27,30^ The Supplementary Material includes an infographic on the associations between outcomes and early mobilization after hip fracture surgery.

**Table I.** Summary of studies investigating associations of outcomes with early/late mobilization in hip fracture care.

Outcome (measure)	Association with mobilization (Early: within 48 hrs after surgery vs late: > 48 hrs)			Number of studies showing positive association with early mobilization
	**Negative**	**No association**	**Positive**	
**Patient outcomes (measures)**				
Improved functional status at various timepoints (mILOA, modified Barthel Index, Harris Hip Score, Merle d’Aubigné score)		^12^	^11^,^22^^r^,^24^,^**26**^^**r**^	4/5
Improved first mobilization (timed to sit on bed, bed to chair)			^21^,^22^^r^,^29^	3/3
Improved mobility status in acute care (Timed Up and Go, time to stand, time to walk, ability to walk/ambulate)	^29^	^12^	^11^,^**13**^,^17^,^18^,^20^,^22^^r^,^24^,^**26**^^**r**^,^27^,^30^	10/12
Improved mobility post discharge (30 day, 0 to 2 mths, 2 to 6 mths; subscales of the FIM, freely mobile without aids, mobile outdoors with one aid, mobile outdoors with two aids or frame, some indoor mobility but never goes out)			^17^,^21^,^**23**^,^28^	4/4
Increased patient commitment/engagement with mobilization (patient participation, accepted physiotherapists assessments, high frequency PT/OT, more time spent in physiotherapy, low assistance requests)		^29^	^17^,^**22**^^**r**^,^**26**^^**r**^,^**30**^	4/5
Better QoL at 12 wks and at 6 mths postop (EQ-5D, VAS/Index, SF-12, pain score at 1 mth postop)	^**11**^	^12,22^ ^r^	^24^	1/4
**Clinical outcomes (measures)**				
Decreased major/minor adverse events/complications in acute care (needed ICU services, morbidity, delirium, pneumonia, pulmonary embolism, pressure ulcers, UTI, fixation failure, DVT, acute kidney injury, anaemia, transfusion, sepsis/septic shock, respiratory failure, cardiac arrest)	^24,29^		^**12**^,^15^,^**16**^,^**18**^,^22^^r^,^**30**^	6/8
Decreased total length of hospital stay (includes acute inpatient rehabilitation)	^24,26^ ^r^		^11^,^12^,^13^,^**14**^,^15^,^**16**^,^**18**^,^19^,^**22**^^r^,^29^,^30^	11/13
Discharged to their pre-injury residence/home	^29^		^13^,^**14**^,^**15**^,^**16**^,^17^,^22^^r^,^26^^r^,^30^	8/9
Fewer hospital readmissions within 1 mth			** ^**16**^ **,^18^	2/2
Fewer hospital readmissions within 3 mths			^**16**^	1/1
Fewer hospital readmissions within 6 mths			^22^ ^r^,^28^	2/2
Fewer reoperation within 30 days		^18^		0/1
Decreased in-hospital mortality			^11^,^**12**^,^**16**^,^**25**^,^29^	5/5
Decreased mortality within 30 days postop			** ^**15**^ **,**^**18**^**,^19^,^20^,^**23**^,^**27**^	6/6
Decreased mortality within 6 mths postop			** ^**21**^ **,**^**30**^**	2/2
Decreased mortality within 1 yr postop			** ^**16**^ **,**^**27**^**	2/2

Superscript ‘r’ denotes evidence from randomized clinical trials.

Statistically significant results are shown bold and underlined.

DVT, deep vein thrombosis; EQ-5D, EuroQol five-dimension questionnaire; FIM, Functional Independence Measure; ICU, intensive care unit; mILOA, Modified Iowa Level of Assistance; OT, occupational therapist; PT, physiotherapist; QoL, quality of life; SF-12, 12 item short form survey; UTI, urinary tract infection; VAS, visual analogue scale; WB, weightbearing.

Four out of the four studies which assessed mobility post-discharge from hospital reported a positive association with early mobilization while in hospital,^17,21,23,28^ as measured by outcome measurement tools such as the Functional Independence Measure (FIM) questionnaire^21,28^ or versions of the New Mobility Scale.^28^

Four other studies found mostly positive associations between early mobilization and subsequent assessments of activities of daily living (e.g. Barthel Index), and also health-related quality of life (HRQoL) at 12 weeks and at six months postoperatively (EuroQol five-dimension questionnaire (EQ-5D), visual analogue scale (VAS)/Index, and 12-item short form survey (SF-12).^11,22,24,26,31^

The evidence from both RCTs indicated that early mobilization increased patients’ commitment to mobilization, and improved mobility in acute care and functional status at various timepoints.^22,26^

In total, early mobilization (within 48 hours of surgery) was associated with improved outcomes in 29 of the 33 patient-reported outcomes.

### Outcomes reported from medical records/routine data

The association between early mobilization and complications (‘need ICU services’, delirium, pneumonia, deep vein thrombosis or pulmonary embolism, pressure ulcers, urinary tract infection, acute kidney injury, anaemia, transfusion, sepsis/septic shock, respiratory failure, cardiac arrest, etc.) was investigated by eight studies, and six of these found lower rates of complications in patients who were mobilized early.^12,15,16,18,22,30^

A total of 11 out of 13 studies found that early mobilization was associated with a reduction in length of hospital stay.^11-16,18,19,22,29,30^ Furthermore, eight of nine studies found that patients who mobilized early were more likely to return to their pre-injury residence when they left the hospital.^13-17,22,26,30^

Mortality was measured at various timepoints (in hospital, and at one, six, and 12 months) in a total of 15 studies, of which five found that early mobilization was associated with a lower in-hospital mortality,^11,12,16,25,29^ and six studies found a lower 30-day mortality in those who mobilized early.^15,18-20,23,27^

The evidence from RCTs indicated that early mobilization decreased complications,^22^ length of stay,^22^ and readmissions within six months post-surgery,^22^ and more patients were discharged to their pre-injury residence/home.^22,26^

Overall, 45 out of 51 clinical outcomes derived from hospital records showed a positive association with early mobilization.

## Discussion

This systematic review summarizes the peer-reviewed literature and provides an overview of the association between ‘early mobilization’ and outcomes following hip fracture surgery in older patients. The heterogeneity of study design, poorly described definitions of early mobilization and weightbearing, plus the wide range of outcomes reported, precluded meta-analysis of the data. However, the existing evidence indicates a positive association between early mobilization and better outcomes in both the short and medium term following surgery. This association was identified for both patient-reported outcomes, such as activities of daily living scores and HRQoL, and clinical outcomes assessed from the medical records or routine data, such as length of hospital stay and patients’ ability to return to their pre-injury place of residence. Early mobilization was also associated with lower mortality. Our findings add to the evidence that early, unrestricted weightbearing after surgery for hip fracture in older people is associated with better recovery.

The decision on when to allow unrestricted weightbearing on the hip following surgery is usually informed by the operating surgeon as part of the postoperative plan documented in their operation record.^32^ Previous research shows that surgeons recognize that older patients with a hip fracture often cannot tolerate restricted weightbearing, and that this limits their ability to mobilize but, nonetheless, surgeons may continue to recommend such restrictions.^33^ Reasons expressed for this decision-making include the type of surgery performed, surgeons being more likely to recommend unrestricted weightbearing when performing arthroplasty compared with fixation surgery, concerns about bone density, and prior experience of implant/fixation failure when patients had no restrictions on the weight they put on their operated hip.

However, weightbearing is only one element of mobilization. Even when patients are able to put full weight on their operated leg, they may still struggle to mobilize. Previous research shows that the reasons for late mobilization are multifactorial.^34^ Non-modifiable barriers to mobilization include many patient-related variables, such as age, pre-injury mobility, and cognitive impairment.^35^ However, the role of patients’ and healthcare providers’ sex in hip fracture care, particularly early mobilization, is not fully explored or understood.^36^ Other factors that limit patients’ ability to mobilize after surgery may be modifiable by, for example, reducing the risk of perioperative delirium, or complications such as pressure sores.^37,38^

Non-patient-related factors that influence patients’ mobility after hip fracture surgery include hospital resources, such as the availability of nursing and therapy staff, and the availability of walking aids.^39^ These may be difficult to mitigate, particularly in resource-poor healthcare settings. However, by involving different members of staff in mobilizing patients, some of these resource issues may be overcome.^40^ Staff attitudes regarding the importance of early mobilization are another important factor, and one that is potentially amenable to change, defining staff roles in mobilization appearing to be key.^41,42^

Patient and family buy-in is another important factor in facilitating early mobilization.^43^ Providing patients and family members with information is important.^44^ In particular, reassuring both patient and family that the perceived risk of further falls and injury is outweighed by the risks of delaying mobilization in terms of, for example, reducing the risk of complications such as infection and thrombosis.^45^

This review is not without limitations. The variable terminology used in describing early mobilization, and poor documentation of weightbearing status, made it difficult to develop a comprehensive search strategy. We attempted to mitigate this using ‘snowballing’ methods, which included conducting an extensive review of reference lists from retrieved studies and by searching the associated grey literature, but it is still possible that our literature search missed some potentially relevant studies. The heterogeneity in the definitions of early mobilization and weightbearing in hip fracture care precluded numerical synthesis of data from different studies, and hence the review is limited to a narrative summary. Finally, although the evidence allowed us to identify associations between early mobilization and better recovery from hip fracture surgery, these data should not be taken as proof of causality. The associations observed are likely to be subject to residual confounding at both a patient and healthcare provider level through, for example, differences in pre-fracture functional status, delirium, comorbidities, and healthcare resource availability. Future randomized trials, such as HIPCARE^46^ and WHiTE - INITIATE,^47^ should help mitigate the risk of confounding in evidence syntheses.^48^

In conclusion, the reporting of performance indicators, such as early mobilization after surgery, is increasingly used to drive improvements in care for patients after hip fracture. This evidence synthesis found that early mobilization after surgery for hip fracture in older people is associated with improved patient-reported outcomes and better clinical outcomes, such as reduced length of hospital stay. Standardization of the definition of early mobilization and consistent reporting of weightbearing status would improve future evidence synthesis.

**Take home message**

- Performance indicators, such as early mobilization after surgery, are increasingly used to assess the quality of care provided for patients after hip fracture.

- Early mobilization (within 48 hours of surgery) after hip fracture in older people is associated with better clinical outcomes and improved patient-reported outcomes.

- Standardization of the definition of ‘early’ and consistent reporting of 'weightbearing status' would improve future evidence synthesis.

## Data Availability

All data generated or analyzed during this study are included in the published article and/or in the supplementary material.
